# The Impact of Preeclampsia and Gestational Diabetes on Future Maternal Cardiometabolic Health

**DOI:** 10.1111/apha.70113

**Published:** 2025-09-29

**Authors:** Alice M. Barrell, Amanda N. Sferruzzi‐Perri

**Affiliations:** ^1^ Loke Centre for Trophoblast Research, and Department of Physiology, Development and Neuroscience University of Cambridge Cambridge UK

**Keywords:** diabetes, heart disease, maternal health, mechanisms, pregnancy

## Abstract

**Introduction:**

Pregnancy is a time of significant maternal physiological change to meet the metabolic demands of the feto‐placental unit. In cases of pregnancy complications, mal‐adaptive physiological responses may occur, potentially impacting the health of both mother and fetus. Moreover, some maternal changes may persist beyond delivery. Although the clinical symptoms of preeclampsia (PE) and gestational diabetes mellitus (GDM) usually resolve post‐partum, growing evidence suggests that these conditions confer a lifelong increased risk of cardiometabolic disease in affected women. This review aimed to summarize epidemiological evidence linking PE and GDM to future maternal cardiometabolic disorders, explore potential underlying mechanisms based on animal and small‐scale human studies, and discuss implications for future research and postpartum clinical care.

**Methods:**

Targeted PubMed searches were conducted to search for relevant publications.

**Results:**

Data suggest that pregnancy complications may both reveal an underlying predisposition to cardiometabolic disease and induce lasting physiological changes that contribute to future health risks. Notably, women with a history of PE may have a 3–4‐fold increased risk of cardiovascular disease, while those with prior GDM may face up to a 10‐fold higher risk of developing type 2 diabetes.

**Conclusion:**

Pregnancy offers a valuable window into a woman's future health, presenting a unique opportunity for preventative medicine for up to half of the world's population.

## 1 Background

Preeclampsia (PE) and gestational diabetes mellitus (GDM) are complications of pregnancy thought to occur in 4.6% [1] and 14% [2] of pregnancies worldwide, respectively. However, these prevalence rates may be underestimates and inaccurate due to a lack of widespread screening and differences in diagnostic criteria [3].

PE is a hypertensive disorder of pregnancy characterized by new‐onset hypertension and maternal systemic dysfunction. The International Society for the Study of Hypertension in Pregnancy (ISSHP) recommends diagnosis of PE when gestational hypertension arises along with at least one of the following conditions from 20 weeks' gestation: proteinuria, other maternal end‐organ dysfunction (including neurological complications, pulmonary oedema, hematological complications, acute kidney injury, or liver involvement), or uteroplacental dysfunction (such as placental abruption or fetal growth restriction) [4].

**FIGURE 1:**
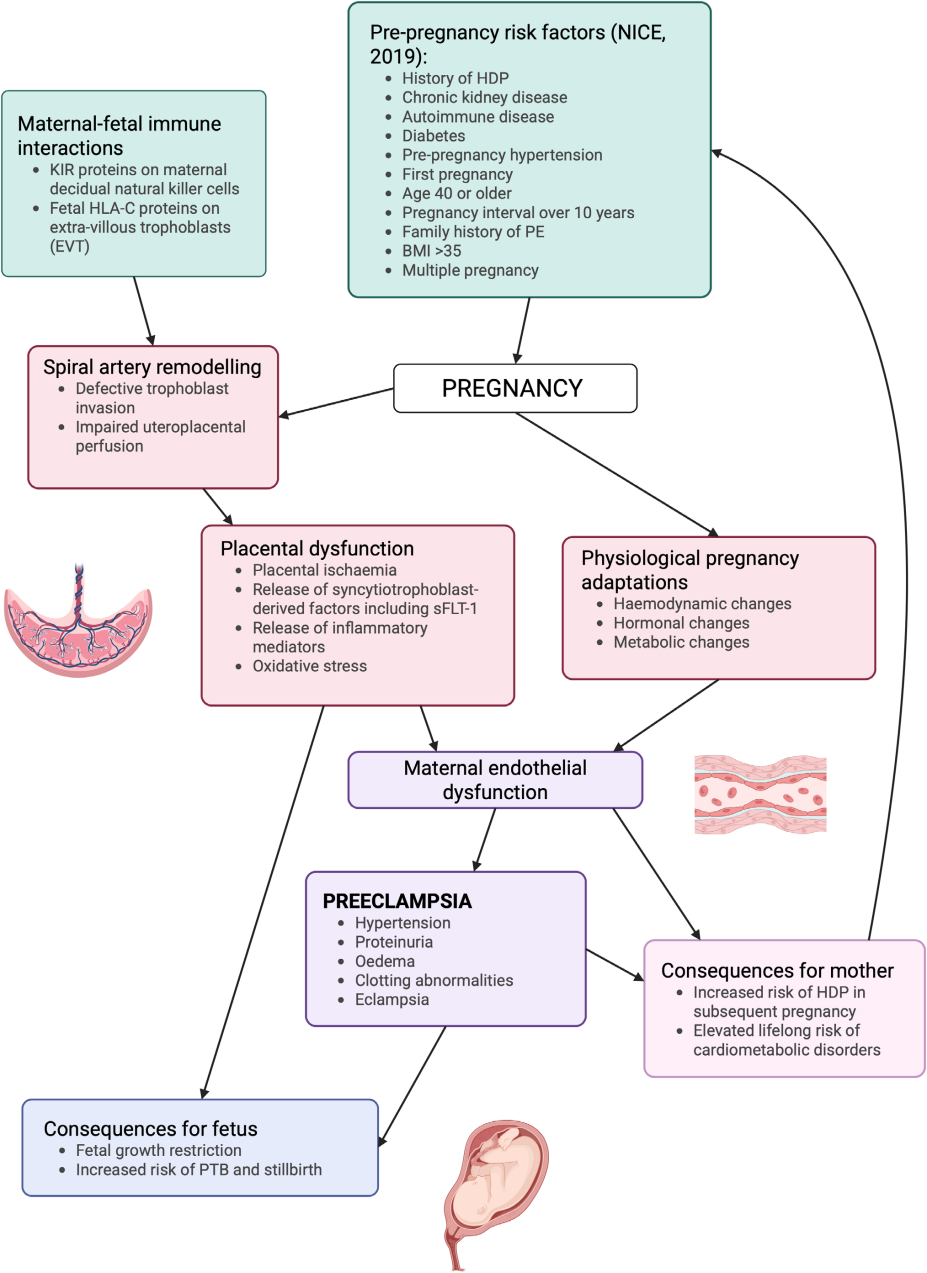
Schematic displaying the pathogenesis of preeclampsia (PE). BMI, body mass index; HDP, hypertensive disorder of pregnancy; KIR, killer‐cell immunoglobulin‐like receptor; PTB, preterm birth; sFlt‐1, soluble FMS‐like tyrosine kinase‐1. Created in BioRender. https://BioRender.com/6ky9mxt.

PE is a multi‐causal disease. Risk factors include a previous hypertensive disorder of pregnancy, pre‐existing chronic illness (such as autoimmune disease, chronic kidney disease, or diabetes), nulliparity, obesity, advanced maternal age, longer inter‐pregnancy interval, family history, and multi‐fetal pregnancy [5] (Figure 1).

PE can be considered an aberrant cardiovascular response to pregnancy. A healthy pregnancy already poses a stress test for the cardiovascular system, creating a hypervolaemic, thrombophilic, and pro‐atherogenic state. However, the exact etiology of PE remains poorly understood, partly due to the lack of spontaneous pre‐clinical models [6].

Classically, PE is divided into early‐ and late‐onset forms. Early‐onset PE is likely caused by aberrant placentation, where failure of maternal uteroplacental spiral artery remodeling by fetal extra‐villous trophoblast cells leads to higher pressure and pulsatile blood flow [7], stressing the placenta. Late‐onset PE was previously thought to be caused by pre‐existing maternal factors, e.g., endothelial dysfunction. However, recent evidence suggests a placental cause for both forms of PE, with the late‐onset form linked to progressive placental mal‐perfusion as fetal demand increases [8]. Both forms likely involve oxidative stress and cell damage in the placenta and the release of factors into the maternal circulation, including fetal cell‐free DNA and pro‐inflammatory cytokines, causing widespread maternal systemic dysfunction (Figure 1).

An emerging biomarker of PE is elevated maternal soluble fms‐like tyrosine kinase 1 (sFlt‐1), an anti‐angiogenic factor released in higher levels from the malperfused, ischaemic placenta. It acts as a decoy receptor for the pro‐angiogenic vascular endothelial growth factor (VEGF) family, including placental growth factor (PlGF), thereby reducing their circulating levels. The sFlt‐1/PlGF ratio is increased in PE [9], contributing to widespread maternal endothelial dysfunction, vasoconstriction, and oxidative stress. Placental dysfunction in PE can also compromise fetal growth (Figure 1).

Gestational diabetes mellitus (GDM) can be seen as another adverse adaptation to the demands of pregnancy. As a healthy pregnancy progresses, mild insulin resistance develops to raise maternal blood glucose, which increases the gradient for trans‐placental glucose transfer to the fetus. This adaptation is facilitated by physiological β‐cell hypertrophy, hyperplasia, and increased glucose‐stimulated insulin secretion, which may occur in anticipation of, or in response to, the increased insulin resistance [10]. In GDM, pathological hyperglycaemia and hyperinsulinaemia most commonly present in the third trimester due to excessive peripheral insulin resistance.

Screening and diagnosis of GDM vary worldwide [11]. The International Association of the Diabetes and Pregnancy Study Groups (IADPSG) diagnostic criteria, accepted by the WHO, recommend diagnosing GDM if fasting plasma glucose > 5.1 mmol/L or 2‐h plasma glucose > 8.5 mmol/L or 1‐h plasma glucose > 10.0 mmol/L [12]. However, these guidelines are not consistently applied across the world. For example, in the UK, a higher threshold for fasting plasma glucose and a lower threshold for 2‐h plasma glucose are used [13].

**FIGURE 2:**
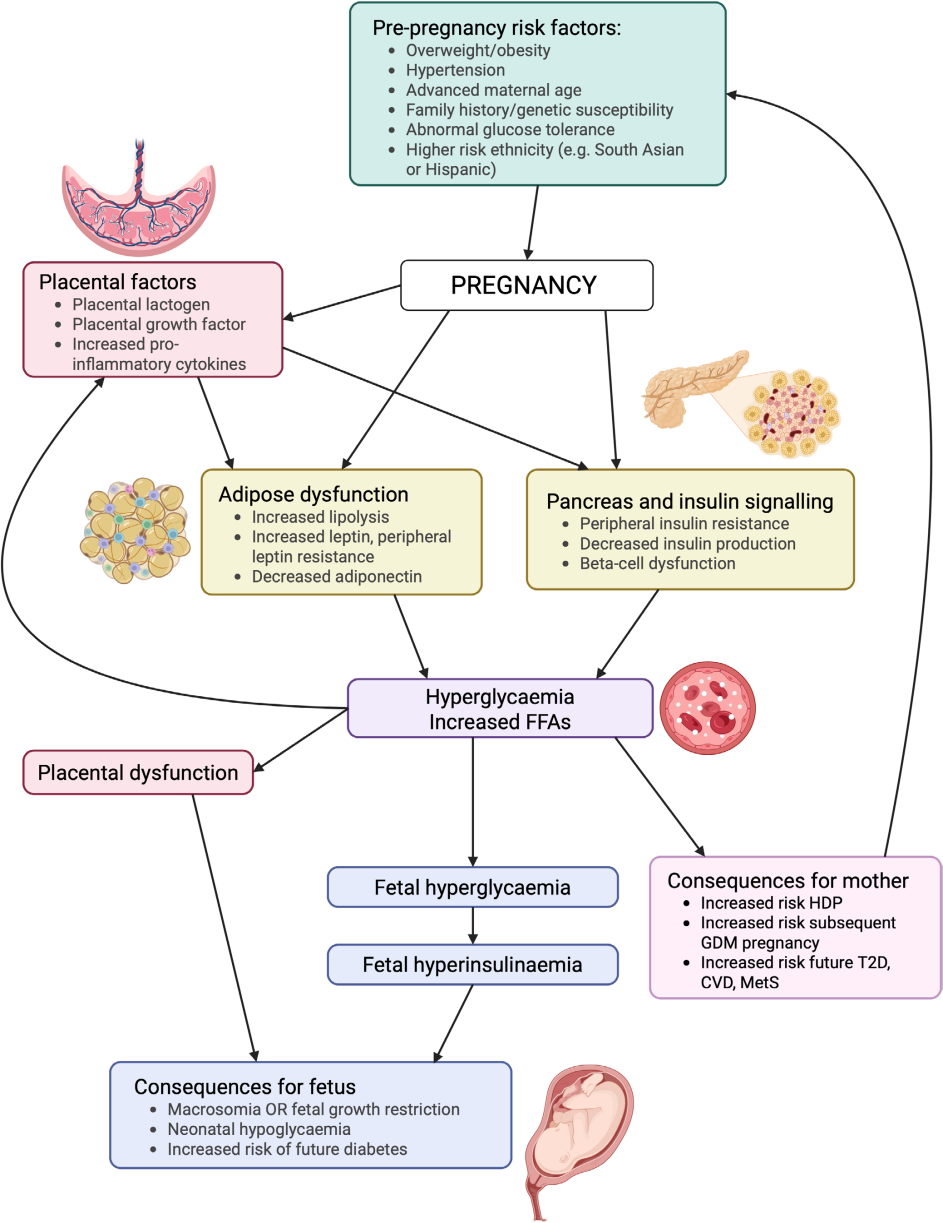
Schematic displaying the pathogenesis of gestational diabetes (GDM). CVD, cardiovascular disease; FFAs, free fatty acids; HDP, hypertensive disorder of pregnancy; MetS, metabolic syndrome; T2D, type 2 diabetes mellitus. Created in BioRender. https://BioRender.com/pgodzvl.

Risk factors for developing GDM include overweight and obesity, a history of GDM in a previous pregnancy, having a baby weighing > 9 lb in a previous pregnancy, a family history of diabetes, certain comorbidities (including high blood pressure, heart disease, and polycystic ovary syndrome), and being of African, South Asian, Native American, or Pacific Island descent [14] (Figure 2).

Since glucose crosses the placenta, GDM can cause fetal hyperglycaemia and, consequently, hyperinsulinaemia. Insulin promotes fetal growth, which can result in a high birth weight and complicated delivery. GDM can also cause fetal growth restriction, likely due to placental dysfunction that impairs nutrient supply to the fetus [15]. It is thought that maternal hyperglycaemia contributes to delayed villous maturation, a placental phenotype characterized by excessive growth and inadequate maturation, increasing the vulnerability of the late gestation fetus to periods of acute hypoxia, which might be a cause of fetal growth restriction and stillbirth [16].

In GDM, hyperglycaemia impairs the ability of β‐cells to sense glucose and release insulin (β‐cell dysfunction), leading to a progressive failure to compensate for peripheral insulin resistance, which in turn worsens hyperglycaemia. After pregnancy, plasma glucose may return to normal, but β‐cell function and peripheral insulin sensitivity might remain impaired, leaving women predisposed to developing type 2 diabetes (T2D).

PE and GDM have many shared risk factors, and GDM itself is a risk factor for PE, even after adjusting for confounders such as pre‐pregnancy body mass index (BMI), maternal age, parity, and pre‐existing chronic diseases, all of which increase the risk of both PE and GDM [17]. This may be due to similar pathophysiological processes in both conditions, including oxidative stress, inflammation, and endothelial dysfunction.

This review aims to investigate the impact of PE and GDM on future maternal cardiometabolic health. First, evidence linking these pregnancy complications with the incidence of later‐life cardiometabolic disease will be summarized. Potential mechanisms underlying this correlation will be discussed, focusing on evidence in human populations and preclinical mouse models. Finally, recommendations for future research and clinical practice will be proposed, given the impact this could have on both individual and population health.

## 2 Pregnancy Complications and Future Maternal Disease—Epidemiological Evidence

To investigate the effect of pregnancy complications on future maternal health, observational studies provide valuable correlative evidence. Evidence linking hypertensive disorders of pregnancy (including PE) to future cardiometabolic disease is presented in Tables 1 and 2, while evidence linking GDM to future cardiometabolic disease is displayed in Tables 3 and 4.

**TABLE 1:** Studies investigating the association between hypertensive disorders of pregnancy (HDPs) and future maternal disease.

Country	Cases	Controls	Follow‐up duration	Exposure	Outcome	Main findings	Adjusted for	Summary	References
Australia	54 323 HDP	490 372	Median 7.3 years	HDP from national records	Hospitalization or death from ischaemic or hypertensive heart disease or stroke, national records	Increased 10‐year risk of CVD following HDP	Age, deprivation, smoking status, GDM, IUGR, preterm birth	HDP ↑ CVD	Arnott et al. 2020 [18]
Canada	6066 recurrent PE 33 493 non‐recurrent PE	567 261	Up to 25 years	PE, hospital records	CVD hospitalization, hospital records	CVD risk is highest for recurrent PE, and higher for non‐recurrent PE than for no PE.	Age, pre‐pregnancy diabetes or CVD, socio‐economic disadvantage, year of delivery	PE ↑ CVD	Auger et al. 2017 [19]
UK	2026 PE 8891 GH	23 937	Up to 57 years	GH: diastolic BP > 90 mmHg PE: GH + proteinuria	Records of CVD hospitalization or death	Similar risk of CVD outcomes with GH or PE, both significantly higher than normotensive pregnancy	Age, smoking status, social class	HDP ↑ CVD	Bhattacharya et al. 2012 [20]
South Korea	3413 PE	65 245	Median 22 years	PE from interview‐based questionnaire	Diagnosis of angina pectoris, MI, or stroke from interview‐based questionnaire	Increased incidence of angina, MI, or stroke following PE vs. normotensive pregnancy	Age, parity, pre‐pregnancy disease, family history of CVD	PE ↑ angina, MI, stroke	Choi et al. 2024 [21]
US	571 HDP	1142	Median 36 years	HDP includes hospital records of PE, GH or chronic hypertension	Medical records of CVD, stroke, or CKD	Significantly increased risk of CVD, stroke, CKD following a history of HDP vs. normotensive pregnancy.	Age, parity, smoking status, pre‐pregnancy BMI, education	PE ↑ CVD, stroke, CKD	Garovic et al. 2020 [22]
Denmark	58 120 PE	1 099 546	At least 35 years	PE from national records	Hospital or death records showing acute MI or ischaemic stroke	Significantly increased risk of MI and stroke following PE vs. normotensive pregnancy	Age, parity, stillbirth	PE ↑ CVD, stroke	Hallum et al. 2023 [23]
Denmark	708 PE	709	Mean 14.5 years	PE from national records	Prevalence of coronary atherosclerosis on cardiac CT scan	Higher prevalence of early coronary atherosclerosis for women with prior PE vs. age‐ and parity‐matched controls	Age, dyslipidaemia, T2D, smoking, BMI, menopause, parity	PE ↑ early coronary atherosclerosis	Hauge et al. 2022 [24]
Denmark	23 330 PE	499 215	Median 10.2 years	PE from national records	Venous thromboembolism from national records	Significantly increased incidence of venous thromboembolism following PE compared to normotensive pregnancy	Age, pre‐pregnancy BMI, known risk factors for venous thromboembolism	PE ↑ venous thromboembolism	Havers‐Borgersen et al. 2023 [25]
Denmark	23 367 PE	500 204	Median 10.1 years	PE from national records	Arrhythmias from national records	Significantly increased incidence of arrhythmias following PE compared to normotensive pregnancy	Smoking status, pre‐pregnancy diseases, known risk factors for arrhythmia	PE ↑ arrhythmias	Havers‐Borgersen et al. 2024 [26]
Norway	21 506 PE with term birth 2649 PE with preterm birth	602 117	Median 13 years	PE +/− preterm birth from hospital records. Preterm birth defined as 16–36 weeks' gestation	Death records for CVD mortality	Higher risk of CVD death following pregnancy with PE and preterm birth, compared to PE with term birth. Both higher CVD deaths than normotensive term birth	Age, year of delivery, birthweight of baby	PE ↑ CVD	Irgens et al. 2001 [27]
Israel	7824 PE	88 546	Mean 11.2 years	PE from hospital records	CVD or renal disease from hospital records	Significantly increased risk of CVD and renal disease hospitalizations following PE pregnancy	Age, parity, pre‐gestational obesity, T2D, smoking status	PE ↑ CVD and renal disease	Kessous et al. 2015 [28]
Finland	1326 PE	843	10 years	PE defined as hypertension + proteinuria after 20 weeks' gestation. Early onset PE < 34 weeks	National records of hypertensive disease	Significantly increased risk of hypertension following PE vs. normotensive pregnancy, highest with early‐onset PE.	Age, parity, pre‐pregnancy BMI, GDM, T2D	PE ↑ hypertension	Kivelä et al. 2023 [29]
UK	25 554	1 277 811	Median 9.25 years	PE from national records	First incidence of CVD *or* CVD death from national records	Significantly increased risk of CVD outcomes following PE vs. normotensive pregnancy	Age, parity, pre‐pregnancy diabetes or hypertension, deprivation index, maternal ethnicity	PE ↑ CVD	Leon et al. 2019 [30]
US	481 PE	13 922	Median 37 years	PE, diagnosed as BP > 140/90 mmHg + proteinuria, after 20 weeks' gestation	Mortality from heart‐related causes, using death records	Significantly increased risk of CVD death following PE vs. normotensive pregnancy	Age, parity, pre‐pregnancy hypertension, socioeconomic status, smoking status, education, IUGR, BMI	PE ↑ CVD	Mongraw‐Chaffin et al. 2010 [31]
Australia	4387 HDP	27 262	26–36 years	PE and GH from hospital records.	Hospital records of admissions for ischaemic heart disease, renal disease, hypertension	Significantly increased risk of hypertension, renal disease, and ischaemic heart disease for either GH or PE compared to normotensive pregnancy	Age, parity, year of delivery	PE ↑ CVD, renal disease	Tooher et al. 2017 [32]
US	128 029 PE	2 404 486	Median 6 years	PE from national records	Hospitalization or death due to heart failure with preserved ejection fraction (HFpEF)	Significantly increased risk of HFpEF hospitalisations following PE vs. normotensive pregnancy	T2D, chronic hypertension, other relevant demographic factors	PE ↑ heart failure	Williams et al. 2021 [33]

*Note:* Controls = women with history of pregnancy without PE. Unless specified, exposure ‘PE’ refers to hospital records using International Classification of Disease (ICD) codes.

**TABLE 2:** Systematic reviews investigating the association between hypertensive disorders of pregnancy (HDPs) and future maternal disease.

Cases	Controls	Outcome	Effect size	References
100 808 PE	2 680 924	Increased risk of end stage renal disease following PE vs. pregnancy without PE	Risk ratio 6.35 (2.73–14.79)	Covella et al. 2019 [34]
598 733 PE	12 563 297	Increased incidence of CVD following PE vs. pregnancy without PE	Effect size: CVD death 2.08 (1.70–2.54) Coronary artery disease 2.04 (1.76–2.48) Heart failure 2.47 (1.89–3.22) Stroke 1.75 (1.52–2.02)	Inversetti et al. 2024 [35]
1 262 726 HDP (including PE, GH)	17 711 054^a^	Increased risk of ischaemic heart disease, heart failure, and hypertension following HDP vs. controls	Relative risk: Ischaemic heart disease 2.06 (1.38–3.08) Heart failure 2.53 (1.28–5.00) Hypertension 3.46 (2.68–4.49)	Sukmanee and Liabsuetrakul, 2022 [36]
72 860 PE	1 961 159	Increased risk of diabetes following PE vs. pregnancy without PE	Adjusted risk ratio: 2.37 (1.89–2.97)	Wu et al. 2016 [37]
258, 275 PE	6 198 104	Increased risk of CVD following PE vs. pregnancy without PE	Risk ratio: Heart failure 4.19 (2.09–8.38) Coronary heart disease 2.5 (1.43–4.37) CVD death 2.21 (1.83–2.66) Stroke 1.81 (1.29–2.55)	Wu et al. 2017 [38]

*Note:* Controls = women with history of pregnancy without PE unless specified.

^a^ Controls includes women with normotensive pregnancy, PE without severe features, or late‐onset PE after 34 weeks of gestation, depending on studies included.

**TABLE 3:** Studies investigating the association between gestational diabetes mellitus (GDM) and future maternal disease.

Country	Cases of GDM	Controls	Follow‐up duration	Exposure	Outcome	Main findings	Adjusted for	Summary	References
US	124	991	25 years	History of GDM, self‐report	NAFLD, measured by CT scan	Increased risk of NAFLD after GDM vs. pregnancy without GDM, significantly attenuated by incident T2D	Age, pre‐pregnancy BMI, pre‐pregnancy metabolic factors, ethnicity	GDM ↑ NAFLD	Ajmera et al. 2016 [39]
Finland	297	297	23 years	GDM, diagnosed by fasting plasma glucose ≥ 4.8 mmol/L and 2 h oral glucose tolerance test ≥ 8.7 mmol/L	T2D, reported in questionnaire	Incidence of T2D: 50.4% after GDM vs. 5.5% after pregnancy without GDM	Cases and controls were pair‐matched for age, parity, and date of delivery	GDM ↑ T2D	Auvinen et al. 2020 [40]
Singapore	142	550	4–6 years	GDM diagnosed by fasting plasma glucose ≥ 7.0 mmol/l and 2 h oral glucose tolerance test ≥ 7.8 mmol/l	T2D diagnosed by fasting plasma glucose ≥ 7.0 mmol/l and 2 h oral glucose tolerance test ≥ 11.1 mmol/l	The highest risk of T2D was seen in women with a history of GDM, pre‐pregnancy overweight, and ≥ 5 kg retained postpartum	Age, ethnicity, education, parity, diabetes family history, HDP	GDM ↑ T2D	Chen et al. 2021 [41]
UK	9118	37 281	Up to 25 years	GDM from primary care records	Ischaemic heart disease, hypertension, T2D from primary care records	Increased risk of T2D or CVD following GDM pregnancy vs. pregnancy without GDM	Age, current BMI, pre‐pregnancy BMI, year of delivery, deprivation, smoking status	GDM ↑ T2D, CVD	Daly et al. 2018 [42]
US	1415	46 056	Mean 10.2 years	GDM, recorded from interview	T2D, recorded from questionnaire	Increased risk of T2D after GDM vs. pregnancy without GDM	Current BMI, pre‐pregnancy BMI, ethnicity, education	GDM ↑ T2D	Diaz‐Santana et al. 2022 [43]
UK	110	113	1–10 years	GDM diagnosed by fasting plasma glucose ≥ 7.0 mmol/L and 2 h oral glucose tolerance test ≥ 7.8 mmol/L	NAFLD diagnosed via ultrasound	Increased risk of NAFLD after GDM vs. pregnancy without GDM	Current BMI	GDM ↑ NAFLD	Forbes et al. 2011 [44]
US	119	779	20 years	GDM recorded from interview, validated with hospital records for a subset of participants	ccIMT measured via ultrasound. T2D diagnosed via fasting plasma glucose and 2 h oral glucose tolerance test and HbA1c	Increased mean ccIMT after GDM vs. pregnancy without GDM. Incidence of T2D: 25% after GDM vs. 6% after pregnancy without GDM	Age, pre‐pregnancy BMI, pre‐pregnancy metabolic factors, ethnicity, parity	GDM ↑ ccIMT, T2D	Gunderson et al. 2014 [45]
Sri Lanka	119	240	11 years	GDM, from questionnaire and patient‐held records	T2D, from medical records. Fasting plasma glucose ≥ 7.0 mmol/L, or 2 h oral glucose tolerance test ≥ 11.0 mmol/L	Incidence of T2D: 61.3% following GDM vs. 5.8% following pregnancy without GDM	Age, family history of T2D, GDM in a previous pregnancy, birth weight, gestational age at delivery	GDM ↑ T2D	Herath et al. 2017 [46]
UK	1390	217 943	Mean 42 years	GDM, from self‐report and medical databases	New occurrence of any CVD outcome aged 40–69 years, from medical databases	Significantly increased risk of CVD after GDM vs. pregnancy without GDM	Age, current BMI, ethnicity, T2D, medication use, menopause status, alcohol use, smoking status	GDM ↑ CVD	Lee et al. 2022 [47]
Canada	67 356	1 003 311	25 years	GDM, from medical records	Hospitalisations for CVD, hospital records	Higher incidence of CVD hospitalization for women with a history of GDM vs. pregnancy without GDM	Age, education, parity, year of delivery, HDP, socioeconomic deprivation	GDM ↑ CVD	McKenzie‐Sampson et al. 2018 [48]

*Note:* Unless otherwise specified, controls = women with a history of pregnancy without GDM. Unless specified, GDM is defined using ICD classifications.

**TABLE 4:** Systematic reviews investigating the association between gestational diabetes mellitus (GDM) and future maternal disease.

Cases	Controls	Outcome	Effect size	References
31 867	643 588	Increased risk of T2D following GDM vs. normoglycaemic pregnancy	Relative risk 7.43 (4.79–11.51)	Bellamy et al. 2009 [49]
310 214	4 155 247	Increased risk of T2D following GDM vs. pregnancy without GDM	Relative risk 8.3 (6.5–10.6)	Dennison et al. 2021 [50]
142 576	3 274 444	Increased risk of CVD following GDM vs. pregnancy without GDM	Odds ratio 1.95 (1.83–2.08)	Li et al. 2018 [51]
67 956	1 264 417	Increased risk of T2D following GDM vs. pregnancy without GDM	Relative risk 9.51 (7.14–12.67)	Vounzoulaki et al. 2020 [52]
2520	3312	Increased risk of metabolic syndrome following GDM vs. normoglycaemic pregnancy	Odds ratio 3.96 (2.99–5.26)	Xu et al. 2014 [53]

*Note:* Unless otherwise specified, controls = women with a history of pregnancy without GDM. Unless specified, GDM is defined using ICD classifications.

### 2.1 Hypertensive Disorders of Pregnancy (Including PE)

Data displayed in Tables 1 and 2 suggests a correlation between hypertensive disorders of pregnancy and future cardiovascular disease (CVD). For example, studies revealed an increased risk of ischaemic heart disease [18, 21, 32], thromboembolic events [22, 25], and heart failure [33]. Death from CVD was also found to be higher following a hypertensive disorder of pregnancy compared to a normotensive pregnancy [20, 31].

In a population cohort study, Leon et al. found that women who developed PE had a 2‐fold increased risk of 12 cardiovascular disorders, with the risk peaking within the first year post‐partum [30]. In a separate population, Hallum et al. found an increased risk of acute myocardial infarction and stroke 20 years after a PE pregnancy [23]. The relative risk of ischaemic cardiovascular disease was highest in women aged 30–39 and within a decade of a PE pregnancy. Hence, the long‐term persistence of this elevated risk suggests permanent changes to the cardiovascular system.

Women with early‐onset PE were 10 times more likely to be diagnosed with hypertension within ten years after pregnancy compared to those with normotensive pregnancies, while women with late‐onset PE had a 7‐fold increased risk [29]. However, the study did not find an elevated risk of other cardiovascular diseases in women with prior PE, unless PE was superimposed on a background of pre‐pregnancy hypertension.

This may be due to the study's shorter follow‐up period, or the younger average maternal age compared to other studies. Notably, the women with superimposed PE tended to be overweight or obese before pregnancy, highlighting the importance of adjusting for pre‐pregnancy BMI when evaluating the association between PE and future maternal cardiometabolic health.

Auger et al. and Kessous et al. found an increased risk of CVD following recurrent PE [19, 28]. Women with recurrent PE had a shorter mean time to cardiovascular hospitalization compared to those with non‐recurrent or no PE over a 25‐year follow‐up period. Recurrent PE may expose women to higher levels of pro‐inflammatory cytokines and oxidative stress, potentially contributing to CVD [19]. Irgens et al. reported that the risk of death from CVD was 8 times higher in pregnancies complicated by both PE and preterm birth (PTB) compared to healthy pregnancies with term delivery [27]. These data may reflect the severity of PE, as early‐onset or severe PE is more likely to lead to PTB compared to less severe or late‐onset PE. These findings suggest that women with recurrent PE, or those with both PE and PTB, may particularly benefit from CVD screening.

In a large cohort study, women with a history of PE had a significantly increased long‐term risk of cardiac arrhythmias, concurrent with hypertension being a well‐established risk factor for arrhythmias [26]. However, whether the underlying mechanisms responsible for this association are the same is not yet elucidated. The study found that women with pre‐pregnancy hypertension had a higher risk of arrhythmias than those with *de novo* PE, suggesting that PE may unmask a population of women with subclinical cardiovascular risk factors. Importantly, the association between arrhythmias and prior PE persisted even after adjusting for multiple confounding factors, supporting the notion that PE is an independent risk factor.

In a case–control study, women with a history of PE had a higher prevalence of early coronary atherosclerosis detected with cardiac CT scanning compared to age‐ and parity‐matched controls [24]. This association remained after adjusting for various cardiovascular risk factors. However, the study was not designed to ascertain whether early coronary atherosclerosis would progress to overt coronary artery disease over time. Future research should investigate this progression to enhance clinical utility.

Data in Table 2 presents evidence from meta‐analyses, supporting the overall finding of increased risk of disease in later life following a hypertensive disorder of pregnancy. Studies found an increased risk of CVD [35, 36, 38], renal disease [34] and diabetes [37]. However, these papers noted substantial heterogeneity in effect sizes across all measured outcomes, likely due in part to confounders such as age, BMI, and other pre‐pregnancy risk factors, which were not adjusted for across included studies.

According to the Preeclampsia foundation, women with a history of PE may have a 3–4‐fold increased risk of developing CVD. Thus, while the evidence confirms an association between hypertensive disorders of pregnancy, including PE, and an increased risk of CVD, it does not robustly quantify the strength of this association, nor establish causation.

### 2.2 Gestational Diabetes

Most of the included studies exploring the impact of GDM and later life health risks have focused on its link to type 2 diabetes (T2D) (Table 3). This was also reflected by various meta‐analyses finding a significant association between GDM and future T2D [49, 50, 52]. Of note, Vounzoulaki et al. (2020) reported women with a history of GDM had up to a 10‐fold increased risk of T2D [52].

In a prospective cohort study of 391 women with GDM and age‐ and parity‐matched controls, the incidence of T2D after 23 years was 50% in women with prior GDM compared to 5.5% in the control group [40]. Another study found a 25% incidence of T2D in women with prior GDM over 20 years, compared to 6% in controls [45]. This study accounted for pre‐pregnancy risk factors, allowing exclusion of women with pre‐pregnancy hyperglycaemia. While there is a difference in the incidence of T2D between studies, both demonstrate a significantly higher risk in women with a history of GDM. The risk appears to be further elevated in those with multiple pregnancies affected by GDM, possibly due to progressive β‐cell dysfunction or stronger pre‐pregnancy risk factors [43]. A particularly high incidence of T2D following a GDM pregnancy was found in a cohort study of Sri Lankan women, with 61% of women developing T2D within 11 years postpartum, and 28% of women with GDM developing T2D in the first 2 years of follow‐up [46]. The authors suggested that this could be due to a lack of support and follow‐up for women postpartum, linked to a lack of understanding of the implications of GDM beyond pregnancy among clinicians and patients.

Xu et al. reported an increased risk of metabolic syndrome, a group of conditions including impaired glucose tolerance, insulin resistance, hypertension, and abdominal obesity, following GDM in a meta‐analysis [53]. The variability between studies decreased when BMI was adjusted for, highlighting pre‐pregnancy overweight/obesity as a key confounder, as it is known to contribute to both GDM and the development of T2D and metabolic syndrome. Chen et al. found that women with prior GDM had the highest risk of developing T2D if they were overweight before pregnancy and experienced substantial postpartum weight retention, compared to those with GDM and who were lean [41]. Non‐alcoholic fatty liver disease (NAFLD), considered the hepatic component of metabolic syndrome [54], is also associated with a history of GDM [39, 44]. Ajmera et al. found that women with prior GDM were more likely to show signs of NAFLD on abdominal CT scan in middle age compared to women with pregnancies unaffected by GDM [39]. This association was no longer statistically significant when the authors controlled for incident T2D after pregnancy, suggesting that progression from GDM to T2D could mediate the increased risk of NAFLD.

In addition to increasing the risk of T2D, there is evidence that prior GDM also raises the risk of CVD [42, 48, 51]. GDM was found to be associated with increased carotid artery intima‐media thickness, a subclinical indicator of early atherosclerosis, even before the onset of overt T2D or metabolic syndrome [45]. The prospective cohort design allowed for the measurement and adjustment of pre‐pregnancy cardiometabolic parameters (glycaemia, insulin resistance, hypertension, and dyslipidaemia), yet the association remained significant, suggesting GDM is an independent risk factor for early atherosclerosis. These findings are supported by a large prospective UK BioBank study of women aged 40–69, which found that prior GDM was linked to an increased risk of various cardiovascular outcomes [47].

## 3 Proposed Mechanisms

**FIGURE 3:**
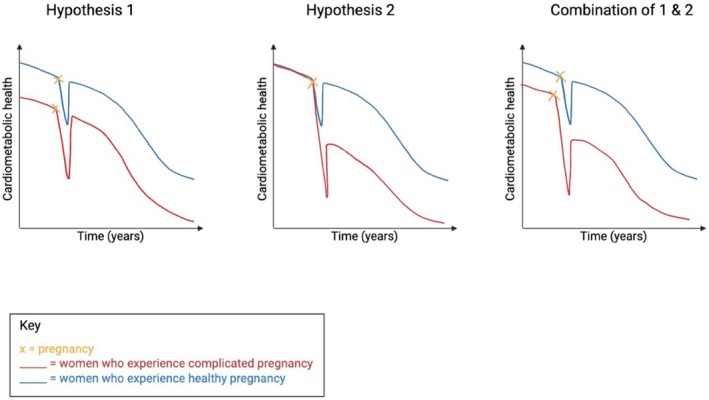
Schematic representing hypotheses underlying the link between a complication during pregnancy and post‐pregnancy poor maternal health. Hypothesis 1: Pre‐existing risk factors and genetics set some women on a trajectory of adverse health, manifesting in *both* complicated pregnancy and future disease, compared to other women with better baseline health, as well as healthy pregnancy. Hypothesis 2: A complicated pregnancy has lasting effects on the health of women, leading to further decline in cardiometabolic health. A combination of hypotheses 1 and 2: Pre‐existing genetic and lifestyle factors, and changes to maternal physiology caused by complications of pregnancy, may cause impaired cardiometabolic health for women in later life.

Evidence from animal models and human populations can help elucidate causal mechanisms linking pregnancy complications with future cardiometabolic disease. Two primary mechanistic hypotheses are as follows (Figure 3):

Hypothesis 1 *Pregnancy acts as a stress test, with complications exposing a population with elevated pre‐pregnancy risk of cardiometabolic disease*.

Hypothesis 2 *Pregnancy complications permanently alter maternal physiology, making them independent risk factors for disease*.

These hypotheses are not mutually exclusive. Both could identify a population at risk, supporting the need for targeted clinical interventions.

### 3.1 Evidence for Hypothesis 1

#### 3.1.1 Shared Genetics

There is evidence of a shared genetic background between some pregnancy complications and future diseases, such as GDM and T2D. In a genome‐wide association study (GWAS) of 5485 women with GDM and 347 856 without, Pervjakova et al. identified five loci significantly associated with GDM, four of which were linked to T2D [55]. One of these loci, *MTNR1B*, encodes MT2, a melatonin receptor implicated in blood glucose homeostasis [56]. However, in a larger GWAS with 12 332 GDM cases and 131 109 parous controls, Elliott et al. found that many genetic risk variants for GDM were independent of T2D and were pregnancy‐specific [57]. Therefore, while there likely exists a set of risk alleles common to both GDM and T2D, further research is needed to clarify the extent of this shared risk. Indeed, a recent study found that British South Asian women with a higher polygenic risk score for T2D and GDM were more likely to develop GDM, and 30% of these women in the highest polygenic risk score decile went on to develop T2D in later life [58]. Taking a genetic approach could thus help to identify which women with GDM are more likely to progress to T2D.

#### 3.1.2 Shared Non‐Genetic Factors

There is also evidence of shared non‐genetic risk factors between pregnancy complications and future diseases. Khan et al. investigated the association between early‐pregnancy BMI, pregnancy complications (including hypertensive disorders of pregnancy and GDM), and postpartum CVD risk factors (hypertension, hyperlipidaemia, and hyperglycaemia) in 4216 women [59]. They found hypertensive disorders of pregnancy mediated only 13% of the relationship between early‐pregnancy overweight/obesity and postpartum hypertension, while GDM mediated 10% of the association between early‐pregnancy overweight/obesity and postpartum fasting glucose levels. These results suggest that GDM and hypertensive disorders of pregnancy may serve as markers of pre‐existing CVD risk, rather than direct causes of postpartum CVD risk. However, the study was limited by its focus on postpartum CVD risk factors, rather than the actual incidence of CVD over time.

**FIGURE 4:**
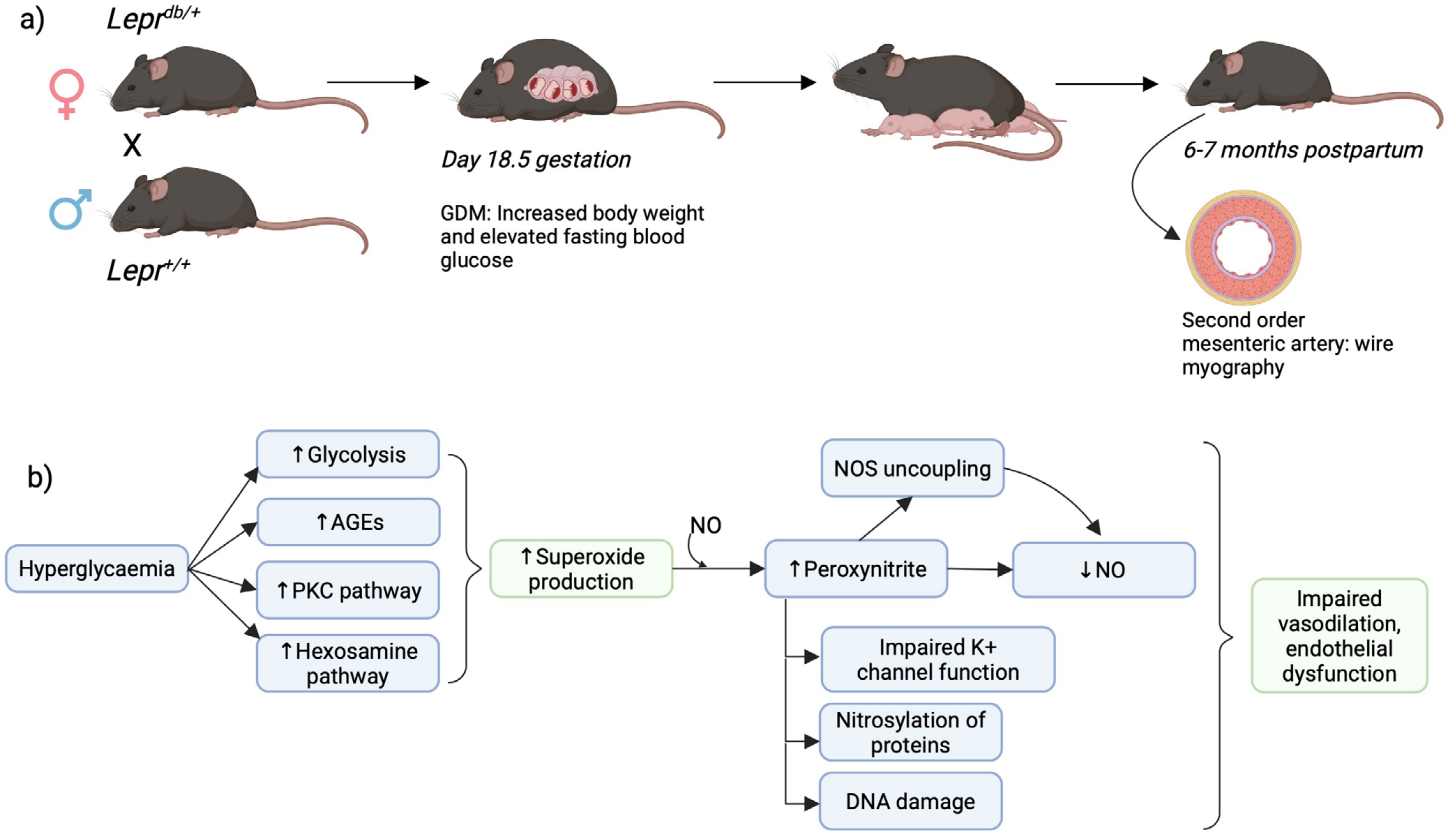
Schematic representation of the mouse model used to understand the link between gestational diabetes (GDM) and post‐pregnancy vascular dysfunction. (a) Method used by Stanley et al. (2010). Female mice heterozygous for leptin receptor gene deletion was mated with wild type (WT) males. This results in the spontaneous development of GDM‐like phenotype in pregnancy, ascertained through weight gain and elevated fasting blood glucose, measured on gestational day 18.5. Mesenteric arteries from mice 6–7 months after pregnancy show endothelial dysfunction. (b) Hyperglycaemia‐induced superoxide overproduction leads to a reduction in the production and bioavailability of nitric oxide (NO). This impairs vasodilation and endothelial function. AGE, advanced glycation end products; NO, nitric oxide; PKC, protein kinase C. Created in BioRender. https://BioRender.com/ze8bau2.

Whether pregnancy complications unmask an underlying pre‐pregnancy disease risk is difficult to demonstrate in cohort studies, as most do not measure pre‐pregnancy factors. Retnakaran and Shah suggested that pre‐pregnancy risk factors could be subtle and sub‐clinical, but still causative in pregnancy complications and future disease [60]. In a cohort of 101 161 women with pre‐pregnancy records, they found that those who developed GDM had higher pre‐pregnancy LDL cholesterol, glucose, and HbA1C (a proxy for average blood glucose levels) than women who did not. These measurements tended to increase in the years leading up to the onset of GDM. Thus, women with GDM were already on a divergent cardiometabolic trajectory, with GDM representing an earlier manifestation of their underlying risk, foreshadowing T2D (as in Figure 4).

Known cardiovascular risk factors may predispose one to PE, which could also explain the postpartum elevated risk of CVD. Elevated pre‐pregnancy serum triglycerides, cholesterol, and higher baseline systolic blood pressure have all been associated with the development of PE in a prospective cohort study [61]. In addition, conditions such as diabetes, chronic hypertension, and obesity are well‐recognized risk factors for developing PE [62]. These conditions are also established risk factors for CVD, making it plausible that a PE pregnancy unmasks these risk factors, revealing women who already have a higher risk of later life CVD. Supporting this, Stuart et al. found that the elevated risk of future CVD events following hypertensive disorders of pregnancy was largely explained by well‐established CVD risk factors in the years after pregnancy; namely hypertension, T2D, hypercholesterolaemia, and elevated BMI [63]. However, it remains unclear whether hypertensive disorders of pregnancy contribute towards the development of these risk factors, as well as unmasking them.

### 3.2 Evidence for Hypothesis 2

There is also evidence that pregnancy complications could permanently alter maternal physiology. Much of this evidence is from animal models because it is possible to remove confounding effects of pre‐pregnancy health, control genetic factors, and perform more invasive investigations.

#### 3.2.1 Persistent Endothelial Dysfunction

Endothelial dysfunction and oxidative stress are components of PE and GDM and may contribute to persistent vascular damage. In a *db/db* mouse model of GDM, Stanley et al. found that *db/db* mice exhibited increased vasopressor responses, decreased endothelium‐dependent vasodilation, and elevated vascular superoxide production 6–7 months after pregnancy [64]. In this model, mice with a heterozygous loss‐of‐function mutation in the leptin receptor spontaneously develop a GDM‐like phenotype during pregnancy (Figure 4a). By using both WT littermate virgin and pregnant controls, Stanley et al. were able to assess the effects of GDM versus normal pregnancy on endothelial function postpartum. In virgin mice, vasodilation in mesenteric arteries was primarily nitric oxide (NO)‐dependent (as inhibition of NO synthase significantly impaired relaxation). However, following normal pregnancy, a non‐NO‐dependent vasodilatory mechanism was upregulated, perhaps reflecting a persistent pregnancy adaptation. This adaptation did not occur in *db/db* mice, which instead displayed an increased reliance on NO for vasodilation post‐pregnancy. A greater dependence on NO‐mediated vasodilation could heighten susceptibility to CVD in later life, as aging is associated with a decline in NO availability in humans [65]. This decline could lead to a greater loss of vasodilation, precipitating hypertension and pressure overload‐induced heart failure. However, this hypothesis would need to be investigated in women following a GDM pregnancy.

The authors suggested that the reason for this impairment in endothelium‐dependent vasodilation could be due to hyperglycaemia‐induced superoxide overproduction (Figure 4b). They found increased levels of superoxide production and peroxynitrite formation in the arteries of GDM mice 6–7 months after pregnancy, despite normal glucose tolerance at this point. Given the reliance on NO‐mediated vasodilation in the post‐GDM mice that was observed, the authors suggested that elevated superoxide production and increased scavenging of NO by peroxynitrite in the arteries might cause a large impairment in endothelial function.

The applicability of Stanley et al.'s findings depends on how representative this model is of GDM in humans. The heterozygous loss of the leptin receptor gene may have additional effects on pre‐pregnancy health that could confound findings, which would make it more supportive of hypothesis 1, if there is a pre‐pregnancy predisposition towards GDM. Additionally, in *db/db* mice, GDM develops due to excess weight gain *during* pregnancy, whereas in humans, pre‐pregnancy obesity is a major risk factor. Moreover, GDM can occur in women without substantial pregnancy weight gain, suggesting that this model might have limited utility in exploring T2D risk in these women.

There is evidence from human studies that GDM and PE might cause persistent endothelial dysfunction, predisposing to CVD. Markers of endothelial dysfunction, such as circulating E‐selectin and intercellular adhesion molecule 1 (ICAM‐1) were found to be elevated in women with a history of GDM 6.5 years after delivery [66]. This association remained significant after adjusting for current BMI and fasting glucose levels. Notably, elevated markers were also present in women with a history of GDM but no current metabolic abnormalities, suggesting a potential clinical risk. However, as the study did not record long‐term CVD incidence, whether these markers translate into an elevated CVD risk remains unknown.

A study investigating endothelial function in small arteries in women 2 years postpartum found that there was impaired endothelium‐dependent vasodilation in arteries from women with prior GDM and subclinical impaired glucose tolerance compared to controls [67]. Inhibition of NO synthase significantly reduced endothelium‐dependent vasodilation in controls, but not in arteries from women with GDM or impaired glucose tolerance, which suggested that a mechanism causing endothelial dysfunction after GDM could be impaired NO synthase activity.

Not all studies have found significant differences in endothelial function between women with and without a history of GDM. Brewster et al. assessed flow‐mediated dilatation, a non‐invasive measure of endothelial function using inflation of a blood pressure cuff to stimulate upstream arterial dilatation, in 117 women 6 years after a normoglycaemic or impaired glycaemic pregnancy [68]. They found no difference in endothelial dysfunction between the groups. In contrast, some studies investigating flow‐mediated dilatation in the immediate postpartum period have found impaired endothelial function in women with prior GDM [69, 70]. This discrepancy suggests that endothelial dysfunction may improve with time and may not be the mediator of elevated CVD risk in women with a history of GDM. Conflicting findings could also be due to the small sample sizes across studies or differences in inclusion criteria for GDM.

However, there is some evidence for endothelial dysfunction persisting after a PE pregnancy, which could mediate some of the elevated CVD risk. A systematic review and meta‐analysis found that women with PE had reduced flow‐mediated dilatation before, during, and after diagnosis, with impairment persisting for at least 3 years postpartum [71]. However, the authors highlighted several limitations, including small sample sizes and a lack of data on pre‐pregnancy risk factors for PE, making it difficult to ascertain whether endothelial dysfunction preceded or resulted from PE. Variability in flow‐mediated dilatation measurement techniques further complicates the interpretation of findings.

#### 3.2.2 Increased Susceptibility to Vascular Injury

**FIGURE 5:**
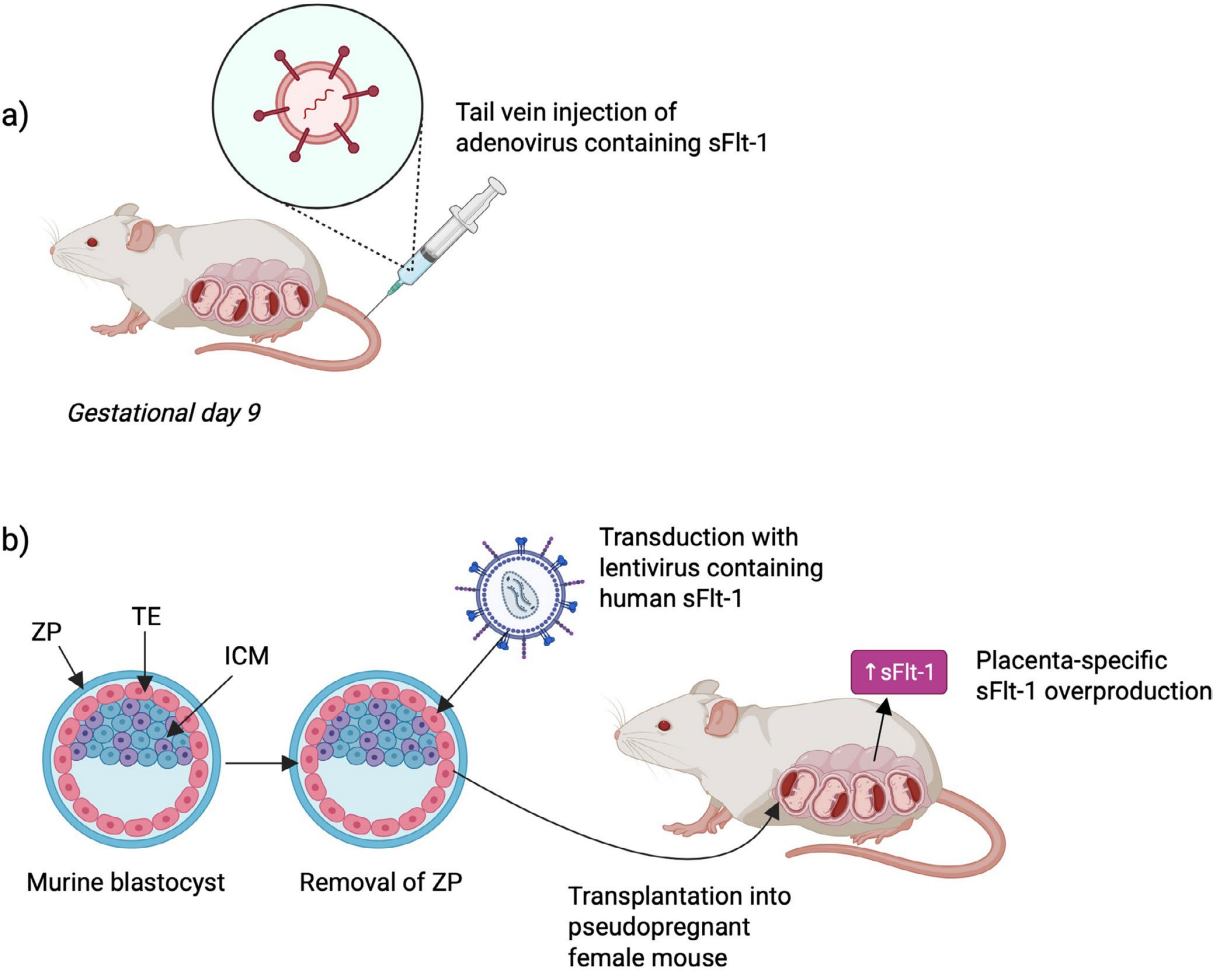
Schematic representation of mouse models used to study the pathogenesis and post‐pregnancy impacts of preeclampsia (PE). (a) Technique used by Pruthi et al. to induce sFlt‐1 overexpression in a mouse model of PE. On gestational day 9, an adenoviral vector carrying sFlt‐1 gene is injected. (b) Technique used by Kumasawa et al. to induce sFlt‐1 overexpression in mouse model of PE. Murine blastocyst first has zona pellucida (ZP) removed and is then transduced with a lentivirus carrying hsFlt‐1 which specifically infects the trophectoderm (TE) lineage without infecting the inner cell mass (ICM). This leads to placenta‐specific overexpression of sFlt‐1 when the blastocyst is transferred to a pseudopregnant female mouse. Created in BioRender. https://BioRender.com/qsp4ugl.

In a murine model, Pruthi et al. suggested that PE may increase susceptibility to future vascular injury [72]. A PE‐like phenotype was induced by overexpressing the anti‐angiogenic factor sFlt‐1, using an adenoviral vector injected into the maternal circulation on gestational day 9, corresponding to the end of the first trimester [9] (Figure 5a). As with human PE, blood pressure and sFlt‐1 levels normalized after pregnancy. However, adverse effects of prior PE became apparent when mice were challenged with carotid vascular injury 2 months after delivery; mice with prior PE had significantly more vascular remodeling and fibrosis compared to controls. The authors proposed a “two‐hit” hypothesis, suggesting that if these findings translate to humans, CVD risk following PE may be exposed or exacerbated by additional risk factors acquired with aging, such as chronic hypertension or atherosclerosis. It is possible that increased sFlt‐1 during pregnancy causes persistent vascular damage, which could be explored in human studies by investigating whether PE cases with higher sFlt‐1 levels are associated with worse CVD outcomes.

With refined methodology, this model could be further used to investigate the mechanisms by which sFlt‐1 affects the maternal vasculature. In this model, systemic adenoviral vector administration resulted in sFlt‐1 production primarily in the maternal liver, whereas in human PE, sFLT‐1 is mainly derived from the placenta [9]. Kumasawa et al. developed a placenta‐specific model, whereby the murine blastocyst was transduced with a lentivirus carrying human (h)sFlt‐1, leading to trophectoderm‐lineage‐specific hsFlt1 overexpression [73] (Figure 5b). This model recapitulated many key features of human PE, including impaired placentation, mid‐gestation hypertension, and proteinuria that resolved postpartum. However, whether this model also replicates the increased long‐term CVD risk remains to be determined through follow‐up postpartum studies.

#### 3.2.3 Renin‐Angiotensin System (RAS)

The renin angiotensin system (RAS) is involved in blood pressure control, water and sodium homeostasis, and vascular tone. In a healthy pregnancy, reduced sensitivity to angiotensin II (Ang II) reduces vasoconstriction, decreasing systemic vascular resistance [74]. However, in PE, Ang II sensitivity is increased during pregnancy and appears to persist postpartum. Stanhewicz et al. showed that within a year postpartum, women with prior PE exhibited greater microvascular vasoconstrictor responses to Ang II compared to age‐matched controls [75]. This was supported by their finding of increased Ang II type‐1 receptor (AT1R) protein levels in their microvasculature following PE. Persistently heightened Ang II sensitivity could contribute to the elevated risk of CVD after PE. However, determining causation would require measuring Ang II sensitivity before pregnancy.

Agonist antibodies at the angiotensin 1 receptor (AT1R‐AA), which promote vasoconstriction, are also known to be present in maternal circulation during PE [76]. Minhas et al. detected these antibodies up to 4 years postpartum, even before signs of cardiac dysfunction [77]. However, this study was limited by a small cohort size (21 women with prior PE and 20 age‐ and BMI‐matched normotensive controls) and the absence of pre‐pregnancy measurement of AT1R‐AA, making it unclear whether these antibodies were a cause of PE or preceded it. Larger cohort studies are required to determine whether AT1R‐AA is directly associated with CVD incidence.

Booz et al. found circulating AT1R‐AA in rats during a PE‐like pregnancy using the reduced uterine perfusion pressure (RUPP) model [78]. In this model, clips are placed around the abdominal aorta and uterine arcades to mimic placental malperfusion in PE. Inhibiting AT1R‐AA during pregnancy increased antioxidant capacity and improved postpartum blood pressure. If these benefits translate to humans, AT1R‐AA inhibition could be explored as both a treatment for PE and a preventative strategy for maternal CVD. However, rigorous pregnancy safety evaluations would be required.

#### 3.2.4 Persistent Cardiac Changes

It could be speculated that pregnancy complications induce lasting maternal cardiovascular remodeling, contributing to the elevated CVD risk seen in women with a history of GDM [79] and PE [23].

Findings from a transgenic rat model [80] suggest that PE can lead to permanent cardiac remodeling, predisposing individuals to CVD. Female rats expressing the human angiotensin gene were mated with males expressing the human renin gene, resulting in a PE‐like phenotype during pregnancy. Postpartum, echocardiography revealed a reduction in ejection fraction and a > 10% increase in left ventricular mass, accompanied by upregulation of genes associated with hypertrophy, fibrosis, and inflammation. The role of RAS overactivity in mediating these changes warrants further investigation. While this model artificially raised RAS activity in pregnancy, it is plausible that AT1R‐AA presence in women with PE could similarly drive long‐term cardiovascular changes.

There is growing evidence in women suggesting impaired cardiovascular function after PE. A multicentre cohort study of 321 women investigated left atrial mechanics using echocardiography 6 months postpartum, finding that prior PE was associated with increased left atrial stiffness and reduced left atrial reservoir strain, regardless of persistent hypertension [81]. These indices resembled those typically seen in women 20–30 years older, suggesting accelerated cardiovascular aging. Left atrial strain is a known predictor of all‐cause mortality in women [82]. However, longer‐term follow‐up is required to determine whether postpartum impairments in left atrial strain and stiffness persist and are linked with increased adverse cardiovascular events in women with a history of PE.

Studies from Orabona and colleagues have found arterial stiffness, increased left ventricular mass, impaired myocardial contractility, and cardiac fibrosis in women up to 4 years after PE [83]. However, these studies were based on small sample sizes (30 women with prior PE and 30 controls). The data suggest more severe remodeling for early‐onset PE compared to late‐onset, potentially due to different aetiologies or prolonged exposure to pro‐inflammatory and oxidative conditions. Middle‐aged women with a history of early‐onset PE have been found to have thicker left ventricular walls and worse diastolic function, as assessed by echocardiography; features that often precede heart failure with preserved ejection fraction in older women [84]. These echocardiographic differences persisted even after adjusting for cardiovascular risk factors including age, blood pressure, and BMI, suggesting that PE may be an independent risk factor for impaired myocardial function in midlife.

Melchiorre et al. investigated echocardiographic changes in cardiac function for women a year postpartum and followed up the cohort 2 years postpartum to measure blood pressure [85]. They found a higher incidence of asymptomatic left ventricular dysfunction and hypertrophy in women with a history of PE. The highest incidence followed preterm PE (56%) compared to term PE (14%) and matched controls (8%). Cardiac dysfunction a year postpartum was associated with an increased incidence of developing essential hypertension by two years postpartum, suggesting that persistent cardiac changes following a PE pregnancy could underlie the elevated risk of hypertension following PE. 70% of women with prior preterm preeclampsia met the criteria for stage B (asymptomatic) heart failure a year postpartum, which could highlight a group of women at risk of progressing to symptomatic heart failure in later life.

There is also evidence suggesting that GDM may lead to cardiovascular remodeling in women, possibly increasing the risk of CVD. In a longitudinal study, women with a history of GDM had greater left ventricular mass, impaired left ventricular relaxation, and reduced systolic function compared to women with a history of normal pregnancy [86]. These changes could be early markers of cardiovascular dysfunction. However, it remains unclear whether they are mediated by pre‐pregnancy obesity and accelerated weight gain during pregnancy, or by the direct effects of hyperglycaemia and inflammatory mediators on cardiomyocytes and endothelial cells. Of note, this association persisted independently of the development of T2D. Similarly, a recent study found that 8–10 years postpartum, women with a history of GDM had increased left ventricular wall thickness and impaired diastolic function, again independent of current type 2 diabetes status [77]. The study suggested that cardiac remodeling following GDM might be similar to diabetic cardiomyopathy. Larger studies are required to confirm these findings and assess whether these early markers of cardiovascular impairment lead to clinical CVD.

Considering pregnancy as a stress test for the heart could provide an opportunity to predict future CVD. This also supports the hypothesis that cardiovascular changes induced by PE may stem from the same underlying risk profile that contributes to CVD.

#### 3.2.5 Persistent Epigenetic Changes

Epigenetics studies gene expression changes that occur independently of the DNA base sequence. These include DNA methylation, histone modifications, and non‐coding RNAs such as microRNAs (miRNAs), which usually prevent target mRNA expression or translation. Environmental stressors can induce epigenetic changes, leading to altered gene expression. It is biologically plausible that complicated pregnancy could induce epigenetic changes, potentially resulting in a persistently elevated risk of cardiometabolic diseases.

Hromadnikova et al. investigated miRNAs associated with CVD pathogenesis in the blood of women with a history of hypertensive disorder of pregnancy (*n* = 275 women) [87] and GDM (*n* = 200) [88] up to 11 years postpartum. Specific miRNAs linked to CVD were found to be upregulated in women with prior hypertensive disorders of pregnancy and GDM. However, whether this represents a mechanistic role in the development of future CVD is not known. As miRNAs were not measured before pregnancy, it is unknown whether hypertensive disorders of pregnancy or GDM caused these changes. It would be valuable to follow up women to see whether certain miRNA profiles associated with PE or GDM predict CVD in later life. Such findings could suggest the potential for using blood miRNA profiles as a screening tool for CVD risk.

DNA methylation may also be altered by complicated pregnancies, serving as an epigenetic “memory”. Mousa et al. found differential methylation at several CpG sites in the omental vasculature collected during caesarean‐section delivery of women with PE (*n* = 22) compared to those with healthy pregnancy (*n* = 16) [89]. The most hypomethylated gene in PE women was *TBXAS1*, which encodes an enzyme involved in thromboxane production. Thromboxane promotes hypercoagulability and hypertension and is elevated in maternal blood during PE [90]. The hypomethylation of *TBXAS1* was associated with a functional increase in thromboxane synthase expression in omental fat arteries in preeclamptic women [91]. However, these findings were correlative, and the cell types where methylation changes occurred were not identified. This could be clarified using single‐cell DNA methylation sequencing techniques, such as single‐cell bisulfite sequencing. Further investigation is needed, including the measurement of methylation status before and after pregnancy in different maternal tissues, which could be clinically challenging if it requires surgery for omental biopsy. This would help determine whether the *TBXAS1* hypomethylation is caused by PE and persists beyond pregnancy, as well as whether it is linked with future CVD risk.

There is some evidence for shared epigenetic marks between GDM and T2D. Linares‐Pineda et al. looked at DNA methylation at sites in the genome associated with T2D, in blood samples of pregnant women with or without GDM [92]. They found that a DNA methylation risk score for T2D was associated with having a GDM pregnancy, with the association persisting after adjusting for age, pre‐pregnancy BMI, family history of diabetes, and smoking status. This could suggest that DNA methylation might be shared between GDM and T2D; however, whether GDM causes the change in DNA methylation, or whether it was present pre‐pregnancy, is not known.

### 3.3 Summary of Hypotheses 1 and 2

**FIGURE 6:**
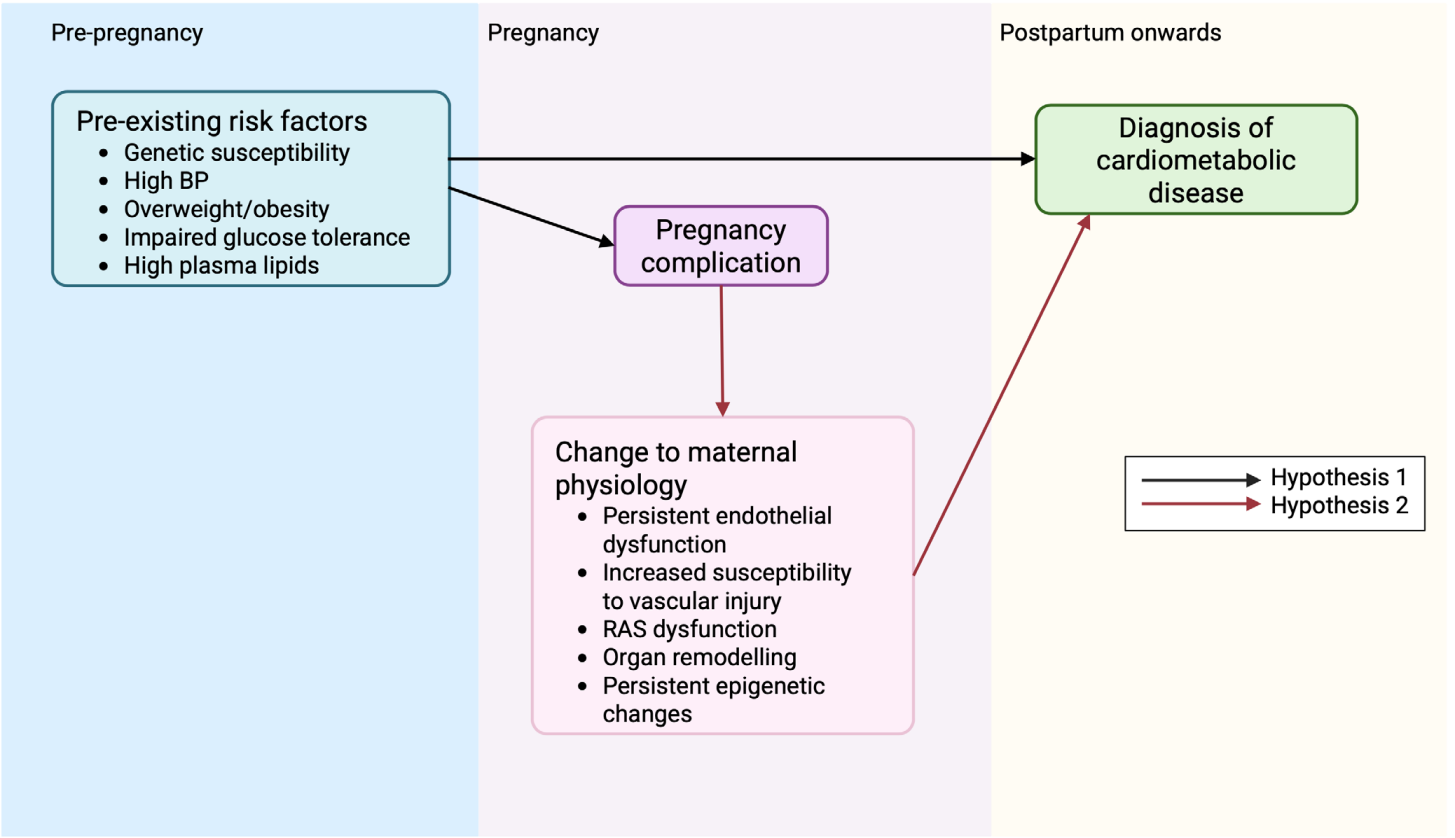
The relationship between pre‐pregnancy, pregnancy and post‐partum maternal health. Black arrows indicate Hypothesis 1, that pregnancy complications reveal a pre‐existing risk of disease. Red arrows indicate Hypothesis 2, that pregnancy complications cause permanent changes to maternal physiology which increase the risk of future disease. Both hypotheses are likely involved. Created in BioRender. https://BioRender.com/ypwx896.

Overall, it is likely that both pre‐existing risk factors and changes induced by pregnancy complications contribute to the lifelong elevated risk of disease (Figure 6). Further research is needed to address the remaining questions regarding the underlying mechanisms. However, the existing correlative epidemiological evidence, along with initial mechanistic findings, suggests a clinically relevant association between pregnancy complications and future cardiometabolic diseases, even before full mechanistic details are elucidated.

## 4 Key Next Steps

### 4.1 Research Recommendations

To better establish whether PE and GDM are independent risk factors for future maternal diseases, or merely markers of a pre‐existing risk, longer‐term cohort studies starting before pregnancy are needed. These studies should ascertain pre‐pregnancy cardiometabolic risk factors such as blood pressure, fasting plasma glucose, and lipids, and BMI. While valuable, such studies present practical limitations. Recruiting participants before pregnancy would require a large sample size to ensure enough women develop PE or GDM and later experience cardiometabolic diseases. Additionally, long‐term follow‐up could result in a high dropout rate.

Animal models can help to elucidate mechanisms, as they allow better control of pre‐pregnancy and genetic factors. Rodents have the benefit of shorter lifespans and the possibility for precise genetic manipulations, enabling the induction of PE or GDM. While they also have a haemochorial placenta, it is less invasive than that of humans, and rodents typically have litters, unlike most human pregnancies [93]. Larger animals may be more physiologically like humans, but their placentas are morphologically different and even less invasive than those of rodents. Additionally, larger animal models come with higher costs and longer lifespans, making long‐term studies more challenging.

For PE research, models are still being refined, e.g., placental sFlt‐1 overexpression, which could provide useful insight given that sFlt‐1 has been suggested as both a biomarker and a causative factor [9]. Previous models [9, 73] used viral vectors to overexpress PE, but these carry the risk of inducing inflammation, which can confound results. More recent developments by Vogtmann and colleagues addressed this issue by utilizing transgenic mouse models to overexpress sFlt‐1 in the placental spongiotrophoblast, which produces endogenous sFlt‐1 in the mouse [94]. The temporally controlled increase in sFlt‐1 mimics key aspects of human PE pathophysiology. Following up mothers from this model after delivery could provide further information on postpartum cardiovascular health.

A combination strategy model could help represent the multi‐causality of pregnancy complications. For example, Li et al. developed a mouse model of lean GDM using a short‐term high‐fat diet (HFD) and low‐dose streptozotocin (STZ) treatment before pregnancy [95]. STZ targets pancreatic β‐cells, permanently impairing insulin secretion, while a low dose induces mild hyperglycaemia. The induced hyperglycaemia resolved after delivery, possibly due to compensation by unaffected β‐cells. Tol et al. used this lean GDM model and found markers of maternal NAFLD postpartum [96], supporting human epidemiological evidence linking GDM to increased risk of NAFLD in later life [39]. This model could also be used to investigate other potential long‐term impacts of GDM, such as CVD and impaired glucose tolerance. Since mice in this model remain lean, it could be particularly useful for studying health outcomes in lean women with GDM. The methodology could be adapted to study obese GDM by extending the duration of HFD feeding.

### 4.2 Challenges and Recommendations for Clinical Practice

Given the evidence linking PE and GDM to future cardiometabolic disorders, and emerging mechanistic findings, pregnancy could serve as a valuable window into a woman's long‐term health.

In the UK, primary care practitioners are advised to discuss the increased risk of future disease following a complicated pregnancy, especially CVD after hypertensive disorders of pregnancy and T2D following GDM [13]. Women with a history of GDM should be offered a fasting plasma glucose test or HbA1c test and are advised to have annual blood glucose screening [13]. Similarly, the American Diabetes Association recommends that women with a history of GDM should be screened for T2D at least every three years [97].

However, research suggests that long‐term follow‐up rates for GDM are low, representing a missed opportunity for diabetes prevention. A survey of 915 randomly sampled English GP practices found that only 39% of practices actively recalled women for annual blood glucose monitoring after GDM, while 35% advised women to arrange follow‐ups themselves [98]. Since these figures are based on GP practice self‐reports, actual figures may be even lower. Around a fifth of practices struggled to identify patients with a history of GDM, and there was confusion over whether primary care or obstetrics departments were responsible. Requiring women to request their T2D screening may pose an additional barrier, disproportionately affecting those from marginalized backgrounds or with greater caring responsibilities, thereby exacerbating health inequalities. A New Zealand cohort study found that just over half of women with a history of GDM received T2D screening within 6 months postpartum, but rates were significantly lower in Māori women and those with higher levels of deprivation [99]. This mirrored disparities in antenatal GDM screening for these groups.

Studies have investigated potential barriers to postpartum T2D screening. A qualitative synthesis of 16 papers representing the 746 women identified key challenges, including difficulty in attending appointments due to work and childcare, a lack of awareness about the risk of progression of GDM to T2D, and fear of a T2D diagnosis [100]. However, it is not clear whether these findings are representative of marginalized patient groups. Future studies should focus on recruiting underrepresented women, including those of low socioeconomic status or from ethnic minority backgrounds, to better understand their specific barriers to screening.

Healthcare provider recommendations may play a crucial role in improving screening rates. A study in Singapore found that women were more likely to complete a postpartum oral glucose tolerance test if it was recommended by a clinician [101]. This suggests that continuity of care and good relationships between women and clinicians could be a facilitating factor. However, this could further widen healthcare inequalities if women from marginalized communities do not feel supported by healthcare providers.

It is important to consider challenges in implementing postpartum follow‐up for women with GDM in lower‐income countries. Effective follow‐up relies on proper GDM screening, yet a study found that this is hindered by limited access to antenatal care, the cost of screening, a lack of knowledge about GDM, and absence of uniform guidelines [102]. Any interventions aimed at reducing the progression of GDM to T2D would require sufficient resources to support women in the postpartum period and beyond, which may not be feasible in all healthcare settings.

In an attempt to address some of these global challenges, the FIGO (International Federation of Gynecology and Obstetrics) Committee on the Impact of Pregnancy on Long‐term Health has developed a pregnancy passport which can be given to women following a complicated pregnancy before they are discharged from hospital. This aims to improve women's awareness of their individual cardiometabolic disease risk, promoting healthy behaviors, as well as prompting healthcare professionals to monitor women's health for the first 12 months after delivery [103].

While screening for T2D is recommended after GDM, there is less guidance about CVD screening following PE, despite its recognition as a risk factor [104]. There is wide variation between national guidelines regarding follow‐up recommendations for PE worldwide [105]. In the UK, guidelines advise women with hypertensive disorders of pregnancy to discuss their increased CVD risk with a GP post‐pregnancy [5], but there is no explicit recommendation for long‐term follow‐up or routine CVD screening. In contrast, The Netherlands and Australia recommended postpartum CVD risk screening for women with a history of PE, while the US and Norway limit screening to those with more severe or recurrent PE [105]. Guidelines could be extended to include more regular cardiovascular monitoring, such as blood pressure, cholesterol, and glucose checks after a hypertensive disorder of pregnancy. PE should be considered a first cardiovascular event, rather than an isolated pregnancy disorder. This shift would better enable interdisciplinary collaboration, with clinicians across specialties routinely enquiring about the pregnancy history in any woman presenting with CVD.

**FIGURE 7:**
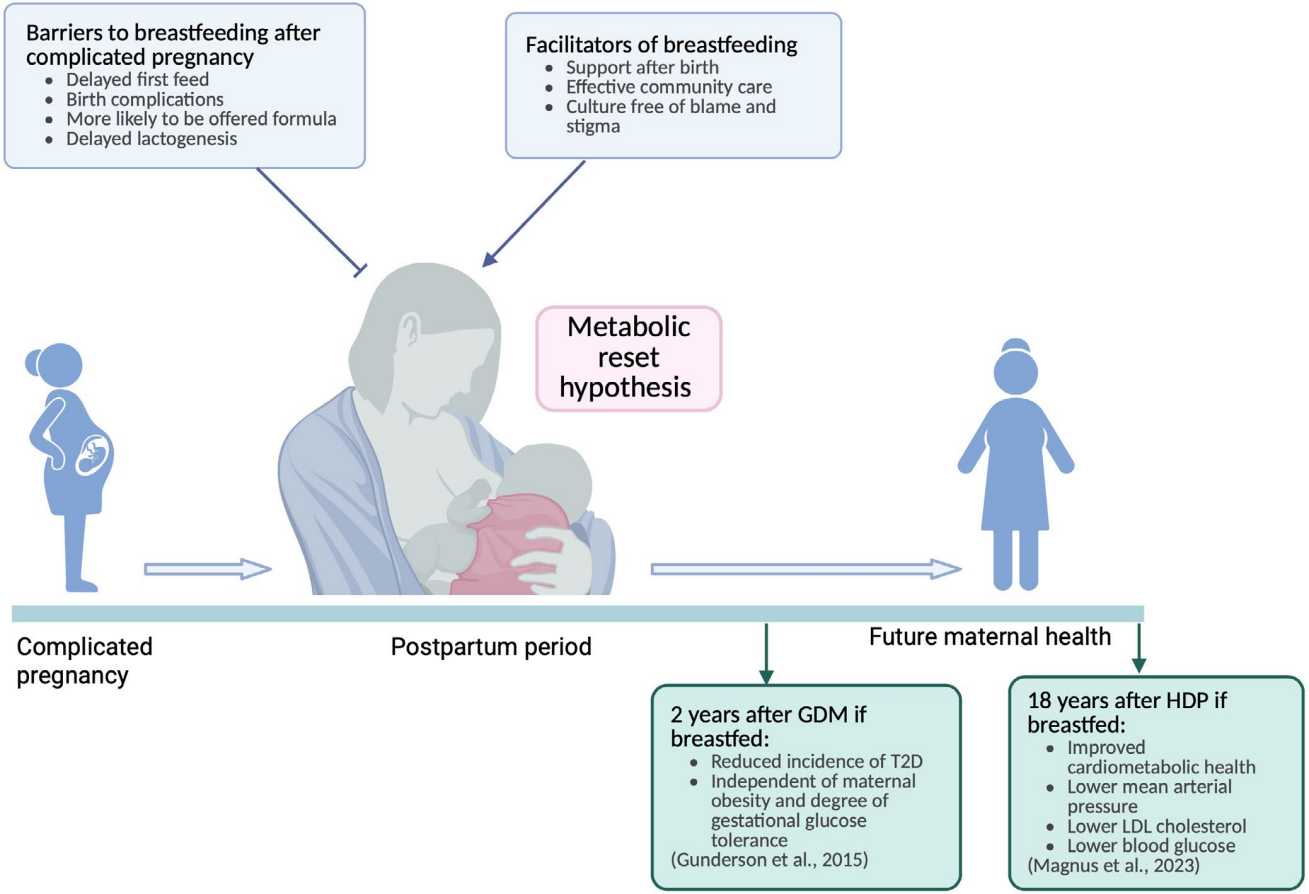
Schematic showing how breastfeeding may offer an opportunity to improve maternal health after a complicated pregnancy. Various factors can either facilitate or hinder successful breastfeeding. Created in BioRender. https://BioRender.com/v66j02e.

Postpartum care represents an opportunity to promote breastfeeding, which appears to confer cardiometabolic benefits after both healthy and complicated pregnancies (Figure 7). A meta‐analysis of over 1 million women found that breastfeeding was associated with a reduced risk of CVD and stroke. However, there was heterogeneity between studies, which may be linked to variations in breastfeeding duration, intensity, and self‐reporting [106]. Gunderson et al. investigated the impact of breastfeeding on the progression of GDM to T2D, finding that 2 years after pregnancy, lactation intensity and duration were associated with a lower incidence of T2D, independent of obesity [107]. Similarly, Magnus et al. reported that breastfeeding was linked to improved cardiovascular health 18 years after a hypertensive disorder of pregnancy, particularly lowering blood pressure, LDL cholesterol, and blood glucose [108]. The authors suggested this could be explained by the reset hypothesis [109], which suggests that fat stores accumulated during pregnancy are mobilized during lactation, promoting a metabolic shift towards the pre‐pregnancy state. Magnus et al. also proposed that oxytocin release during breastfeeding might enhance vascular homeostasis and lower postpartum blood pressure [108].

While breastfeeding appears beneficial, the mechanisms underlying these effects, particularly after complicated pregnancies, remain unclear. Social factors must also be considered, as the narrative “breast is best” can be damaging for women unable to do so. Ensuring adequate infant nutrition should be the priority. Women with PE or GDM may also face additional barriers, such as complicated deliveries that can delay skin‐to‐skin contact. Otter et al. found that women with GDM were more likely to experience delayed first feeds, be offered formula milk, and wean earlier [110]. Maternal obesity (a risk factor for GDM) may also impact lobuloalveologenesis and lactogenesis [111], potentially due to leptin's inhibitory effects on the milk ejection reflex or reduced levels of prolactin [112]. Promoting breastfeeding, both in women with a healthy and complicated pregnancy, should focus on removing barriers such as social stigma and providing lactation support by healthcare professionals.

As well as considering a potential protective effect of breastfeeding following a complicated pregnancy, other interventions to mitigate the increased cardiometabolic risk could be investigated further. It is plausible that metformin, which reduces hyperglycaemia in T2D, could be given in the postpartum period to reduce the progression from GDM to T2D. The Diabetes Prevention Programme found that metformin reduced T2D risk following a GDM pregnancy by 40%, while an intensive lifestyle intervention which included physical activity reduced T2D risk in this group by 35% compared to controls [113]. A recent systematic review found that lifestyle interventions including diet, exercise, and breastfeeding could reduce the incidence of T2D following GDM by 24% [114]. For PE, it is plausible that statins, which have cardio‐protective effects and anti‐inflammatory and anti‐oxidant properties, could reduce the risk of progression to CVD [115]. Studies in rodent models of PE have found beneficial effects of pravastatin during pregnancy in improving postpartum cardiovascular function [80, 116]. However, it remains to be seen whether these effects translate to women, whether there are any effects on the fetus (as pravastatin crosses the placenta), or whether prescribing a statin postpartum could be a viable strategy to reduce CVD risk following PE. Further research could also investigate whether women with a history of PE may benefit from a lower threshold for statin initiation in midlife for cardiovascular risk reduction [117].

Given the challenges and opportunities to improve the health of women following pregnancy complications, there could be a role for digital health innovations. mHealth applications, which use mobile devices, are being increasingly utilized in maternal healthcare and have been found to increase interactions with healthcare services and improve the management of conditions such as GDM [118]. However, while there is an opportunity to improve maternal healthcare in developing countries, this relies on widespread access to the internet, personal devices, and literacy skills, of which there are existing disparities [118]. Digital Health has been named as a priority in the recent UK NHS 10‐year plan, including the management of long‐term conditions through an NHS app [119]. This approach could be applied to the follow‐up of women with pregnancy complications over time, which could also improve health literacy and autonomy for women.

Overall, it would be helpful to consider PE and GDM as chronic conditions, rather than isolated diseases of pregnancy, as their effects persist beyond pregnancy. Taking a “life‐course” approach to women's health more generally involves considering biological and social risk factors at each stage of a woman's life, and how they interact to influence health in later life [120]. Applying this concept to pregnancy complications could help to identify women at risk of cardiometabolic disease [121]. This perspective supports long‐term screening interventions and lifestyle advice, such as the benefits of breastfeeding, a healthy diet, and exercise, to reduce the risk of CVD and T2D. As part of this approach, the potential role of the menopause acting as a ‘second‐hit’ could be considered, wherein hormonal and metabolic changes during the perimenopause period may further exacerbate and reveal underlying cardiometabolic diseases years after pregnancy. While the menopause is generally associated with acceleration of cardiometabolic disease risk [122], how this transition affects women with a history of pregnancy complications could be investigated further. Women with other pregnancy complications, such as premature birth, fetal growth restriction, and placental abruption also face an elevated risk of future disease [123]. Adopting a life course approach could therefore provide long‐term benefits for up to half of the global population.

## 5 Conclusion

This review has some limitations. Much of the mechanistic evidence linking pregnancy complications to future maternal diseases comes from small cohort studies, which may be affected by confounders, or from animal models with fundamental physiological differences compared to humans. Epidemiological evidence largely relies on record linkage studies that often do not account for pre‐pregnancy cardiometabolic risk factors, making it difficult to elucidate underlying causal mechanisms. Additionally, there is substantial heterogeneity across studies in how PE and GDM are defined and diagnosed—ranging from self‐reports to hospital records, with varying criteria across healthcare systems. Longitudinal studies may also be affected by changes in diagnostic standards over time. These issues are further compounded by ethnic and racial diversity, variations in GDM and PE phenotypes, and postpartum environmental influences. Finally, unpublished studies and research from different healthcare systems were not included in this review and may limit the generalizability of the findings and contribute to conflicting results.

In conclusion, PE and GDM appear to be associated with an increased risk of future maternal cardiometabolic disease. However, the precise magnitude of this risk and its underlying causal mechanisms remain unclear. Despite this uncertainty, these findings highlight a population of women who may benefit from clinical interventions. While much research focuses on how the pregnancy environment affects offspring, comparatively less attention has been given to the long‐term implications for mothers. This gap is reinforced in clinical practice, where there is limited screening and follow‐up care for women after a complicated pregnancy.

A better understanding of how pregnancy complications contribute to long‐term maternal health risks could inform evidence‐based interventions to prevent disease progression. Investigating the role of pre‐pregnancy risk factors on both pregnancy outcomes and future disease could also support public health initiatives aimed at improving the health of women. Furthermore, research into how pregnancy complications may have lasting effects on the maternal metabolic and cardiovascular systems could guide the development of pharmacological treatments, either during or after pregnancy, that target underlying mechanisms and reduce long‐term disease risk.

## Author Contributions

**Alice M. Barrell:** conceptualization; visualization, writing – original draft preparation; writing – review and editing (supporting). **Amanda N. Sferruzzi‐Perri:** funding acquisition; supervision; writing – review and editing. Both authors reviewed and approved the final version of the manuscript.

## Ethics Statement

The authors have nothing to report.

## Conflicts of Interest

The authors declare no conflicts of interest.

## Data Availability

Data sharing not applicable to this article as no datasets were generated or analysed during the current study.
